# Moderate-to-vigorous physical activity does not improve mortality in type 2 diabetes patients with severe abdominal aortic calcification

**DOI:** 10.1371/journal.pone.0317007

**Published:** 2025-01-09

**Authors:** Chang Sheng, Yacheng Xiong, Pu Yang, Wei Wang

**Affiliations:** 1 Department of Vascular Surgery, Xiangya Hospital, Central South University, Changsha, Hunan, China; 2 Clinical Research Center for Vascular Intervention in Hunan Province, Changsha, Hunan, China; 3 National Clinical Research Center for Geriatric Disorders, Xiangya Hospital, Central South University, Changsha, Hunan, China; Hamasaki Clinic, JAPAN

## Abstract

**Background:**

The impact of moderate-to-vigorous physical activity (MVPA) on all-cause mortality in type 2 diabetes (T2D) patients with severe abdominal aortic calcification (SAAC) remains unclear.

**Methods:**

We analyzed data from the National Health and Nutrition Examination Survey (NHANES) 2013–2014, including T2D patients aged 40 years and older. AAC was assessed using the Kauppila scoring system, with SAAC defined as a score >6. Self-reported MVPA was categorized based on weekly minutes of activity. The weighted Cox regression model was used to investigate risk associations.

**Results:**

Among the weighted sample of 20,328,606 T2D participants, 16.39% had SAAC. SAAC was significantly associated with increased all-cause mortality (HR 2.57, 95% CI 1.52–4.35) after adjusting for confounders. MVPA did not significantly reduce mortality risk in patients with SAAC (HR 1.00, 95% CI 0.40–2.49).

**Conclusion:**

SAAC is a robust predictor of mortality in T2D patients, and MVPA does not improve mortality outcomes in this high-risk group. Future studies should conduct more detailed subgroup analyses to identify the specific indications for MVPA.

## Introduction

Diabetes, the ninth leading cause of death globally, poses a significant public health concern and imposes a substantial healthcare and economic burden [1]. Individuals with diabetes have a 2- to 4-fold higher risk of cardiovascular events and death compared to the general population [2, 3]. Physical activity is crucial in reducing or even eliminating this excess mortality risk [4–6]. Research has robustly confirmed that engaging in moderate-to-vigorous physical activity (MVPA) can reduce the all-cause mortality risk in patients with type 2 diabetes (T2D) [6, 7]. Nevertheless, it is unclear whether this benefit extends to all diabetes patients.

Patients with T2D may experience physical frailty due to aging and cardiovascular complications [8]. Therefore, it is essential they can engage in physical activities without significant physical strain. Abdominal aortic calcification (AAC), a form of vascular calcification common in diabetes patients, is highly prevalent in aging populations [9]. A systematic review revealed that elderly individuals or patients with chronic kidney disease accompanied by AAC have a significantly higher risk of future cardiovascular events and poorer prognosis [10]. Factors like altered bone metabolism, smoking, hypertension, and low osteocalcin levels further increase the risk of AAC in these patients [11]. A study involving African Americans indicates a significant positive association between diabetes and the presence of AAC [12]. It is currently believed that the overexpression of tumor necrosis factor-α, various interleukins, and different cellular signaling pathways in diabetic patients contributes to the development of vascular calcification [13]. We also know that the AAC score is a predictor of all-cause mortality in T2D patients [14]. Currently, it is unclear how MVPA affects all-cause mortality in T2D patients when considering the heterogeneity of AAC.

To optimize the management of physical activity in diabetic patients, we utilized data from the large-scale National Health and Nutrition Examination Survey (NHANES) to investigate the impact of MVPA on all-cause mortality in T2D patients across different levels of AAC. Furthermore, we aimed to determine whether AAC could enhance the prediction and stratification of all-cause mortality risk in T2D patients.

## Methods

### Design and participants

The NHANES is a continuous, large-scale study that collects health data and conducts examinations on scientifically chosen representative samples of the U.S. population. Our study prospectively analyzed the all-cause mortality of T2D patients aged 40 and above surveyed between 2013 and 2014. In NHANES, AAC assessments were conducted among participants aged over 40 years. Participants were queried or measured to obtain a single valid result. Participants were classified as having diabetes if they met any of the following criteria: (1) random blood glucose level of 11.1 mmol/L or higher, (2) fasting blood glucose level above 7 mmol/L, (3) HbA1c of at least 6.5%, (4) blood glucose of 11.1 mmol/L or more after a 2-hour oral glucose tolerance test, (5) current use of hypoglycemic medication, or (6) documented history of diabetes diagnosis. Inclusion criteria required complete survival data, AAC measurements, and relevant demographic variables. We excluded patients treated solely with insulin and under 20 years old, as they were likely to have type 1 diabetes mellitus [15, 16].

### AAC measurements and definition

The extent of AAC was evaluated using the Kauppila scoring system, assigning a score from 0 to 3 for calcification in each of eight segments, resulting in a total score of 24 [17]. Severe abdominal aortic calcification (SAAC) was defined as an AAC score exceeding 6 [18].

### Risk factor measurements

Demographic information was obtained through questionnaires during household interviews. This included gender, age, poverty income ratio (PIR), race, body mass index (BMI), marital status, and education level. Smoking status was categorized as: former smoker, current smoker, never smoker. Alcohol intake status was categorized into four groups: non-drinkers, consuming 1–5 drinks per month, 5–10 drinks per month, and consuming 10 or more drinks per month. Hypertension is defined as having a stable blood pressure of ≥140/90 mmHg, the use of antihypertensive medication, or a history of hypertension. Chronic kidney disease (CKD) stages were defined as follows: Stages 1–2 were identified by the presence of albuminuria with an eGFR of 60 mL/min/1.73 m^2^ or higher; Stage 3 was defined by an eGFR between 30 and <60 mL/min/1.73 m^2^; and Stages 4–5 were defined by an eGFR below 30 mL/min/1.73 m^2^.

Data concerning self-reported MVPA were gathered using the Global Physical Activity Questionnaire [19]. MVPA is defined as activities that notably increase respiration or heart rate, spanning transportation, occupational, and leisure-time domains. To address data distribution, MVPA and its domains were categorized into binary classes: ’none,’ denoting 0 minutes per week, and ’any,’ indicating more than 0 minutes per week [20].

### Definition of all-cause mortality

Baseline data from NHANES 2013–2014 were linked to mortality records sourced from the National Death Index death certificates. We employed a probabilistic matching algorithm to ascertain mortality status.

### Statistical methods

All analyses were conducted using sample weights, clustering, and stratification to ensure nationally representative estimates. Kaplan-Meier survival curves assessed cumulative mortality across two AAC categories. We used a weighted multivariable Cox proportional hazards model, adjusting for age, sex, race, poverty level, BMI, total cholesterol, high-density lipoprotein (HDL), hypertension, alcohol intake, smoking, and chronic kidney disease [21]. Additionally, we categorized T2D based on AAC levels and conducted multiplicative interaction analyses. Discrimination was assessed using the C-statistic, comparing models adjusted for Framingham risk factors with and without AAC [14, 22, 23]. Net Reclassification Index (NRI) evaluated AAC’s incremental discriminative ability compared to the traditional model [24].

## Results

### Study population

Following the application of weights, the study included a total of 20,328,606 participants. Among diabetic patients, the prevalence of SAAC was 16.39%. In patients with T2D aged over 40, those with an AAC score of 0 are predominant (S1 Fig). Table 1 summarizes baseline characteristics stratified by SAAC levels. The weighted mean age was 61.79 ± 11.01 years, with 46.12% female participants. Significant differences (all P < 0.05) were seen within age, race, marital status, smoking status, and kidney condition across SAAC levels.

**Table 1:** Baseline characteristics of people with diabetes by AAC.

Characteristic	Overall N = 20,328,606	No SAAC N = 16,962,886	SAAC N = 3,365,720	*P* value
Age, years	61.79 (11.01)	60.23 (10.68)	69.78 (9.05)	<0.001
Age strata, %				<0.001
40–59	8,350,453.36 (40.66%)	7,877,875.27 (45.87%)	472,578.08 (14.04%)	
60+	12,188,508.45 (59.34%)	9,295,366.48 (54.13%)	2,893,141.97 (85.96%)	
Sex, %				0.472
Male	11,066,051.73 (53.88%)	9,421,163.70 (54.86%)	1,644,888.03 (48.87%)	
Female	9,472,910.07 (46.12%)	7,752,078.05 (45.14%)	1,720,832.02 (51.13%)	
Race, %				0.001
Non-Hispanic White	13,336,416.71 (64.93%)	10,598,108.58 (61.71%)	2,738,308.13 (81.36%)	
Minority	7,202,545.09 (35.07%)	6,575,133.17 (38.29%)	627,411.92 (18.64%)	
PIR	2.85 (1.57)	2.89 (1.58)	2.67 (1.52)	0.400
PIR strata, %				0.454
<1.38	5,040,097.42 (24.54%)	4,195,648.94 (24.43%)	844,448.48 (25.09%)	
≥1.38 and <3.99	9,458,726.18 (46.05%)	7,643,615.13 (44.51%)	1,815,111.05 (53.93%)	
≥3.99	6,040,138.21 (29.41%)	5,333,977.68 (31.06%)	706,160.52 (20.98%)	
Education level, %				0.129
High school degree/equivalency or less	8,777,660.87 (42.75%)	7,147,113.17 (41.64%)	1,630,547.70 (48.45%)	
Some college or associates degree	7,988,664.24 (38.91%)	6,568,360.36 (38.27%)	1,420,303.88 (42.20%)	
College Graduate or above	3,764,701.73 (18.34%)	3,449,833.26 (20.10%)	314,868.47 (9.36%)	
Marital status, %				<0.001
Married/Living with partner	13,639,064.05 (66.41%)	11,854,503.45 (69.03%)	1,784,560.60 (53.02%)	
Single	6,899,897.76 (33.59%)	5,318,738.30 (30.97%)	1,581,159.45 (46.98%)	
BMI, kg/m^2^				0.414
<25.0	2,178,007.70 (10.60%)	1,688,854.52 (9.83%)	489,153.18 (14.53%)	
≥25.0	18,360,954.10 (89.40%)	15,484,387.23 (90.17%)	2,876,566.87 (85.47%)	
Alcohol Intake				0.495
Non-drinker	6,555,996.04 (31.92%)	5,627,924.36 (32.77%)	928,071.69 (27.57%)	
1 to <5 drinks/month	11,878,967.95 (57.84%)	9,803,222.05 (57.08%)	2,075,745.90 (61.67%)	
5 to <10 drinks/month	857,583.68 (4.18%)	806,253.15 (4.69%)	51,330.53 (1.53%)	
10+ drinks/month	1,246,414.13 (6.07%)	935,842.20 (5.45%)	310,571.94 (9.23%)	
Smoking, %				0.001
Never	9,707,692.20 (47.26%)	8,741,174.71 (50.90%)	966,517.49 (28.72%)	
Former	8,211,597.64 (39.98%)	6,228,353.84 (36.27%)	1,983,243.80 (58.92%)	
Now	2,619,671.96 (12.75%)	2,203,713.20 (12.83%)	415,958.76 (12.36%)	
Total cholesterol, mg/dL	181.60 (57.65)	182.46 (60.39)	177.24 (40.84)	0.850
HDL, mg/dL	47.66 (13.63)	47.73 (13.72)	47.31 (13.21)	0.612
Glycated hemoglobin,	7.13 (1.66)	7.14 (1.69)	7.09 (1.48)	0.732
Hypertension, %	14,014,360.10 (68.23%)	11,469,027.40 (66.78%)	2,545,332.70 (75.63%)	0.082
CKD group, %				0.010
No CKD	13,134,391.90 (63.95%)	11,384,796.13 (NA%)	1,749,595.77 (NA%)	
Stages 1–2	3,044,520.41 (14.82%)	2,618,151.69 (15.25%)	426,368.72 (12.67%)	
Stages 3	3,786,042.49 (18.43%)	2,727,713.98 (15.88%)	1,058,328.51 (31.44%)	
Stages 4–5	574,007.00 (2.79%)	442,579.95 (2.58%)	131,427.05 (3.90%)	
MVPA				0.693
Any (> 0 min/week)	13,354,786.65 (65.02%)	11,083,348.36 (64.54%)	2,271,438.29 (67.49%)	
None (0 min/week)	7,184,175.15 (34.98%)	6,089,893.39 (35.46%)	1,094,281.77 (32.51%)	
Transportation MVPA				0.835
Any (> 0 min/week)	3,606,561.86 (17.56%)	3,047,261.24 (17.74%)	559,300.62 (16.62%)	
None (0 min/week)	16,932,399.94 (82.44%)	14,125,980.51 (82.26%)	2,806,419.43 (83.38%)	
Occupational MVPA				0.954
Any (> 0 min/week)	7,105,642.71 (34.60%)	5,918,003.22 (34.46%)	1,187,639.49 (35.29%)	
None (0 min/week)	13,433,319.09 (65.40%)	11,255,238.53 (65.54%)	2,178,080.56 (64.71%)	
Leisure MVPA				0.199
Any (> 0 min/week)	8,946,845.02 (43.56%)	7,155,820.95 (41.67%)	1,791,024.07 (53.21%)	
None (0 min/week)	11,592,116.79 (56.44%)	10,017,420.80 (58.33%)	1,574,695.98 (46.79%)	

Data are presented as mean (SD), n (%), and *P* value.

Analysis conducted: Wilcoxon rank-sum test for complex survey samples; chi-squared test with Rao & Scott’s second-order correction. Abbreviation: PIR = poverty income ratio, BMI = body mass index, HDL = high-density lipoprotein, CKD = chronic kidney disease, MVPA = moderate-to-vigorous physical activity, AAC = abdominal aortic calcification, SAAC = severe AAC.

**Fig 1:**
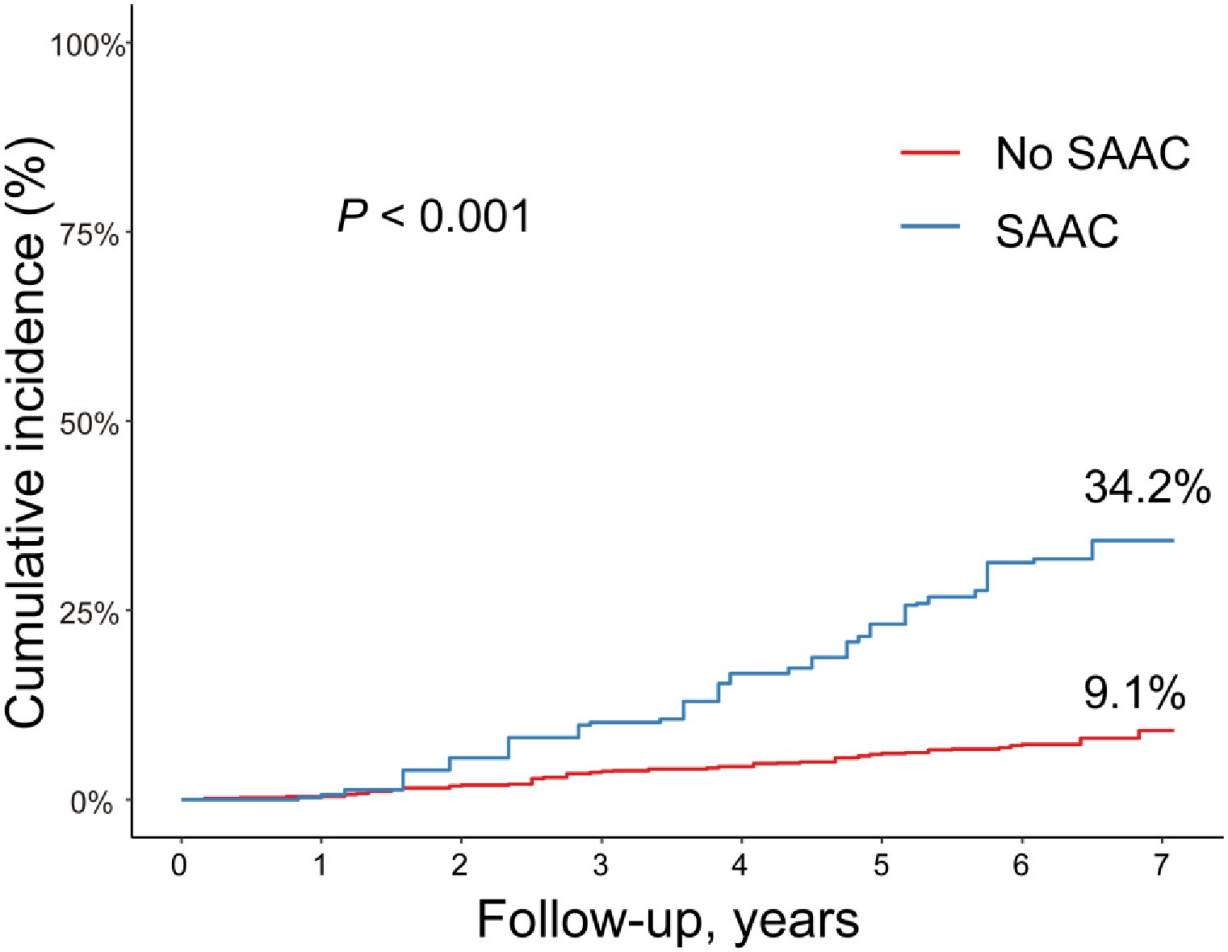
Kaplan–Meier estimates of all-cause mortality events by AAC burden in diabetes patients. Kaplan–Meier curve demonstrating a significantly increased all-cause mortality in patients with SAAC. Abbreviations: AAC = abdominal aortic calcification, SAAC = severe AAC, MAAC = mild–moderate AAC.

Over a median follow-up period of 6.08 years, 2,340,835 deaths occurred (11.40% mortality rate). Weighted death rates for all-cause mortality were 9.1% and 34.2% in the no SAAC and SAAC groups, respectively. The log-rank test revealed a significant association between SAAC and increased cumulative mortality rates in T2D patients (P < 0.001, Fig 1).

### Prediction of all-cause mortality by AAC

In both unadjusted and age/sex-adjusted (A/S adjusted) analyses, SAAC significantly increases the risk of all-cause mortality compared to no AAC. Specifically, the hazard ratio (HR) was 4.62 (95% confidence interval [CI] 3.06–6.97) in the unadjusted analysis and 2.79 (95% CI 1.55–5.02) in the A/S-adjusted analysis (see Table 2). Even after adjusting for multiple variables in the model, SAAC remained significantly increases the risk of all-cause mortality compared to patients without AAC (HR 2.57, 95% CI 1.52–4.35). When analyzing abdominal aortic calcification as a continuous variable, similar results were observed; the multivariate-adjusted HR per 1 standard deviation (SD) increase in calcification scores associated with all-cause mortality was 1.36 (95% CI 1.15–1.61; Table 2).

**Table 2:** HRs per SD of AAC or category of AAC and 95% CIs for regression of all-cause mortality associated with AAC in T2D.

	SAAC categorical		AAC continuous
	No SAAC	SAAC	
Univariable adjusted	1 (Reference)	4.62 (3.06, 6.97), < 0.001	1.68 (1.50, 1.89), < 0.001
A/S adjusted	1 (Reference)	2.79 (1.55, 5.02), < 0.001	1.38 (1.16, 1.65), < 0.001
Multivariable adjusted^a^	1 (Reference)	2.57 (1.52, 4.35), < 0.001	1.36 (1.15, 1.61), < 0.001

Data are presented as HR, 95% CI, and *P* value.

With the exception of AAC categorical, HRs are per 1-SD increase in AAC measures. A/S indicates adjusted for age and sex; Multivariable indicates adjusted for age, sex, race, poverty level, BMI, total cholesterol, HDL, hypertension, alcohol intake, smoking, and CKD.

Abbreviation: HR = hazard ratio, CI = confidence interval, SD = standard deviation, BMI = body mass index, HDL = high-density lipoprotein, CKD = chronic kidney disease, AAC = abdominal aortic calcification, SAAC = severe AAC.

### Discrimination and reclassification for all-cause mortality in diabetes patients

The addition of AAC score significantly improved the discriminatory ability for predicting all-cause mortality over a 6.08-year observation period (C-statistic for the basic model: 0.747; C-statistic after adding AAC score: 0.778; P < 0.05 for the difference between models). The model incorporating AAC score alongside basic variables demonstrated a notable improvement in the net reclassification index (NRI) of 12% (95% CI 7–17%).

### Joint association of AAC and MVPA with all-cause mortality among patients with T2D

Patients with T2D and SAAC face a heightened risk of mortality. We further investigated the combined impact of AAC, MVPA, and all-cause mortality (refer to Table 3 and S2 Fig). Across all subgroups, mortality rates ranged from 2.32% to 41.77%. In the SAAC group, engaging in MVPA weekly did not significantly alter the risk of all-cause mortality compared to not engaging in MVPA weekly, consistent across various MVPA categories.

**Table 3:** The impact of MVPA on all-cause mortality in T2D grouped by SAAC.

Characteristic	SAAC	Cases/total	No SAAC	Cases/total	*P* for interaction
MVPA					0.037
None (0 min/week)	1 (Reference)	457076/1094282	0.64 (0.28, 1.47), 0.293	804942/6089893	
Any (> 0 min/week)	1.00 (0.40, 2.49), 0.998	606787/2271438	0.35 (0.13, 0.94), 0.037	472030/11083348	
Transportation MVPA					0.008
None (0 min/week)	1 (Reference)	831234/2806419	0.52 (0.33, 0.83), 0.006	1206419/14125981	
Any (> 0 min/week)	2.26 (0.64, 7.97), 0.205	232629/559301	0.13 (0.03, 0.59), 0.008	70552/3047261	
Occupational MVPA					0.483
None (0 min/week)	1 (Reference)	806274/2178081	0.42 (0.21, 0.83), 0.013	1058297/11255239	
Any (> 0 min/week)	0.69 (0.28, 1.68), 0.414	257589/1187640	0.63 (0.18, 2.28), 0.483	218675/5918003	
Leisure MVPA					0.070
None (0 min/week)	1 (Reference)	595516/1574696	0.50 (0.25, 0.98), 0.045	1010814/10017421	
Any (> 0 min/week)	0.92 (0.43, 1.97), 0.832	468348/1791024	0.41 (0.16, 1.07), 0.070	266158/7155821	

Data are presented as HR, 95% CI, and *P* value.

HRs are versus the SAAC and none MVPA group. Multivariable indicates adjusted for age, sex, race, poverty level, BMI, total cholesterol, HDL, hypertension, alcohol intake, smoking, and CKD.

Abbreviation: BMI = body mass index, HDL = high-density lipoprotein, CKD = chronic kidney disease, MVPA = moderate-to-vigorous physical activity, SAAC = severe abdominal aortic calcification.

The data also revealed an interaction between AAC and transportation-related MVPA in influencing all-cause mortality in T2D (P for interaction = 0.008). Compared to T2D patients with SAAC who do not engage in transportation MVPA weekly, those engaging in transportation MVPA showed an 87% reduction in mortality risk if they did not have SAAC.

## Discussion

This study investigates how AAC predicts all-cause mortality. It also examines the impact of MVPA on mortality in patients with T2D and SAAC. In a nationally representative sample, we found that AAC is an independent predictor of all-cause mortality and significantly improves prediction beyond traditional risk factors. Additionally, we found that engaging in transportation, occupational, and leisure forms of MVPA does not improve all-cause mortality risk in patients with T2D and SAAC.

In our study, each SD increase in AAC was associated with a 36% higher risk of mortality in T2D patients. In a study conducted by Cox et al. involving approximately 700 T2D patients, it was observed that during an 8.4-year follow-up period, each SD increase in AAC led to a 39% increase in risk [14]. Our study, which included a larger sample and more comprehensive adjustments, reinforces the findings of Cox et al. Similarly, their results also showed that AAC score improved risk prediction and risk reclassification in diabetic patients. Comparisons with other studies are limited. Most research focuses on coronary artery calcification, with few studies addressing AAC or outcomes beyond mortality. However, we reinforced this finding by showing that, in a nationally representative sample, AAC is also a strong predictor of mortality in diabetic patients. The incidental finding of SAAC in T2D patients without evident cardiovascular risk factors may warrant further cardiovascular diagnostic testing. Numerous epidemiological studies have robustly established a link between AAC and adverse cardiovascular events, including, coronary heart disease [25], stroke [26], and myocardial infarction [27]. This may be an underlying mechanism for the observed association. A significant advantage of the AAC score is that it uses an affordable and widely available imaging modality, and its clinical significance can be further explored.

Current practice guidelines encourage T2D patients to engage in consistent physical activity and minimize sedentary behavior, which has multiple benefits [28, 29]. Patients with diabetes commonly present with elevated blood glucose and blood pressure levels, which can lead to vascular endothelial dysfunction, a known risk factor for the onset or exacerbation of cardiovascular disease. Exercise training, particularly aerobic and combined exercise, benefits endothelial function [30, 31], leading to decreases in triglycerides and elevates in high-density lipoprotein (HDL) levels [32]. For patients with T2D, MVPA should be performed without significant physical burden. Due to age and cardiovascular complications, patients with T2D may experience physical frailty [8]. In T2D populations over 40 years old, SAAC constitutes a significant portion. Our study found that T2D patients with SAAC do not benefit from MVPA in terms of all-cause mortality. The reason behind this result may be that the benefits of MVPA are outweighed by the acute physical burden it causes in such populations.

It is noteworthy that our study provides a larger and more diverse sample, thereby enhancing the generalizability of the results. Stratified analysis showed that MVPA affects how AAC influences mortality. This underscores the importance of considering SAAC when studying MVPA’s effects. Future research should explore the mechanisms behind these variations and develop corresponding prevention strategies. The prospective design of our study enhances the credibility of the observed associations.

However, limitations include the potential for residual confounding and the exclusion of certain population segments, which also highlights the need for cautious interpretation. The population with SAAC is of advanced age, and a portion of deaths may not be attributable to SAAC. Additionally, our analysis did not account for dietary habits and psychological states, which might introduce a degree of bias into the results. MVPA information was self-reported, which may involve recall bias. Given that this study is a large-scale investigation, using accelerometers to record objective data may not be feasible. Moreover, self-reported MVPA serves as an effective approach to capturing long-term activity patterns. Additionally, due to data limitations, we did not account for variations in MVPA dosage. Unfortunately, due to the study’s reliance on single baseline measures of MVPA, we were unable to assess the time-dependent association of exposure variables.

This information has the potential to assist in treatment decisions, increase patient awareness of disease risks and symptoms, motivate lifestyle changes, improve individual risk prediction, and identify new targets for innovative treatments. For T2D individuals with SAAC, more tailored MVPA management may be required. Moreover, future research should explore whether understanding AAC enhances primary prevention and clinical management strategies.

## Conclusion

In conclusion, in this nationally representative sample, AAC emerges as a robust predictor of mortality, contributing to enhanced risk assessment and stratification in diabetic care. Additionally, T2D patients with SAAC need to improve all-cause mortality through factors other than moderate-to-vigorous physical activity.

## Supporting information

S1 Fig Distribution of AAC scores in patients with T2D. AAC = abdominal aortic calcification.
(DOCX)

S2 Fig The Impact of MVPA on all-cause mortality in T2D Grouped by SAAC. MVPA = moderate-to-vigorous physical activity, SAAC = severe abdominal aortic calcification.
(DOCX)
