# Supplementary material for: Moderate-to-vigorous physical activity does not improve mortality in type 2 diabetes patients with severe abdominal aortic calcification
Source: PLoS One. 2025 Jan 9;20(1):e0317007. doi: 10.1371/journal.pone.0317007 (PMC11717319; doi:10.1371/journal.pone.0317007)
Supplement: S1 Fig — AAC = abdominal aortic calcification. (DOCX) [file pone.0317007.s001.docx]

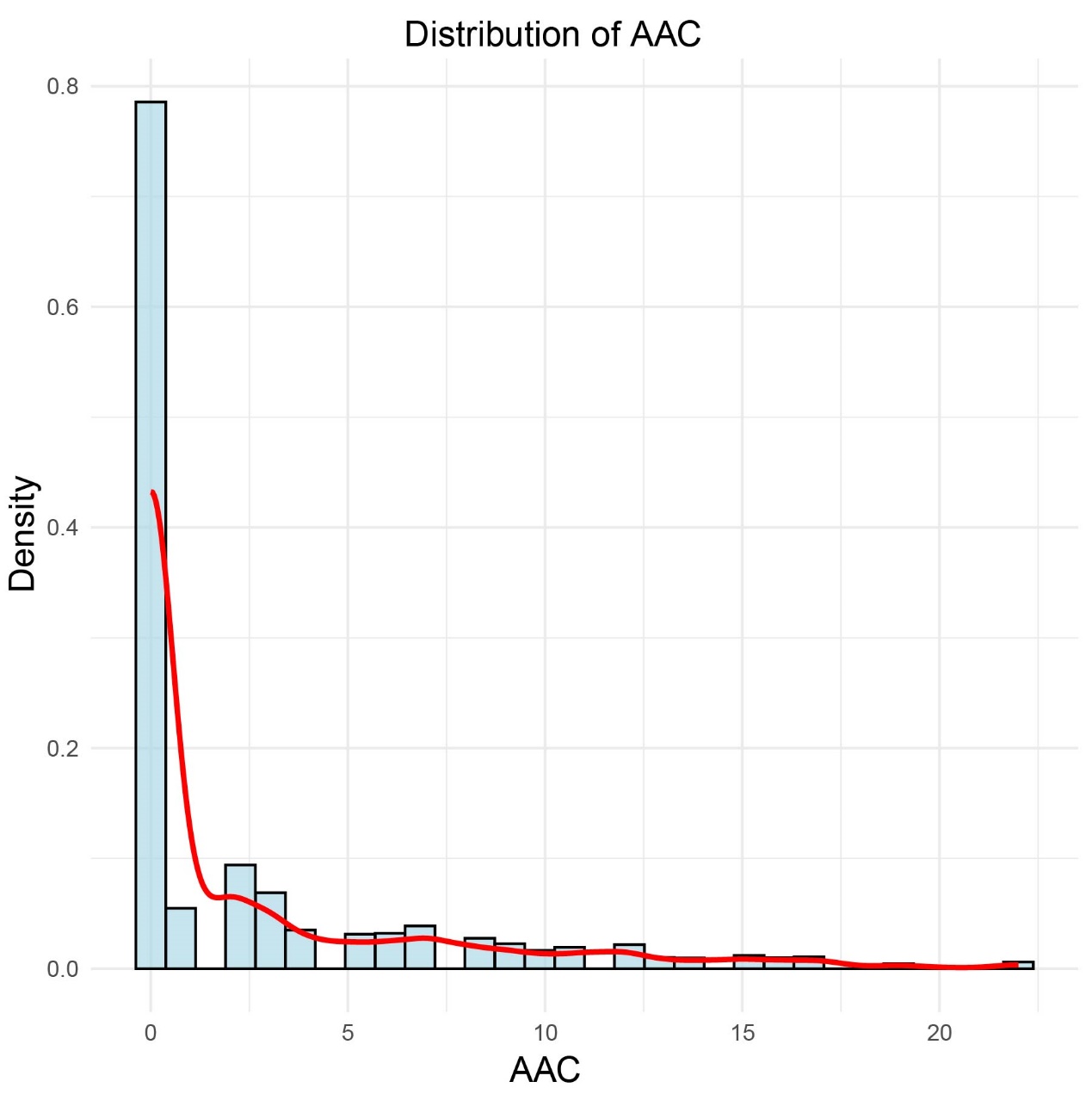


Supplementary Figure S1. Distribution of AAC scores in patients with T2D. AAC = abdominal aortic calcification.
