# Supplementary material for: Moderate-to-vigorous physical activity does not improve mortality in type 2 diabetes patients with severe abdominal aortic calcification
Source: PLoS One. 2025 Jan 9;20(1):e0317007. doi: 10.1371/journal.pone.0317007 (PMC11717319; doi:10.1371/journal.pone.0317007)
Supplement: S2 Fig — MVPA = moderate-to-vigorous physical activity, SAAC = severe abdominal aortic calcification. (DOCX) [file pone.0317007.s002.docx]

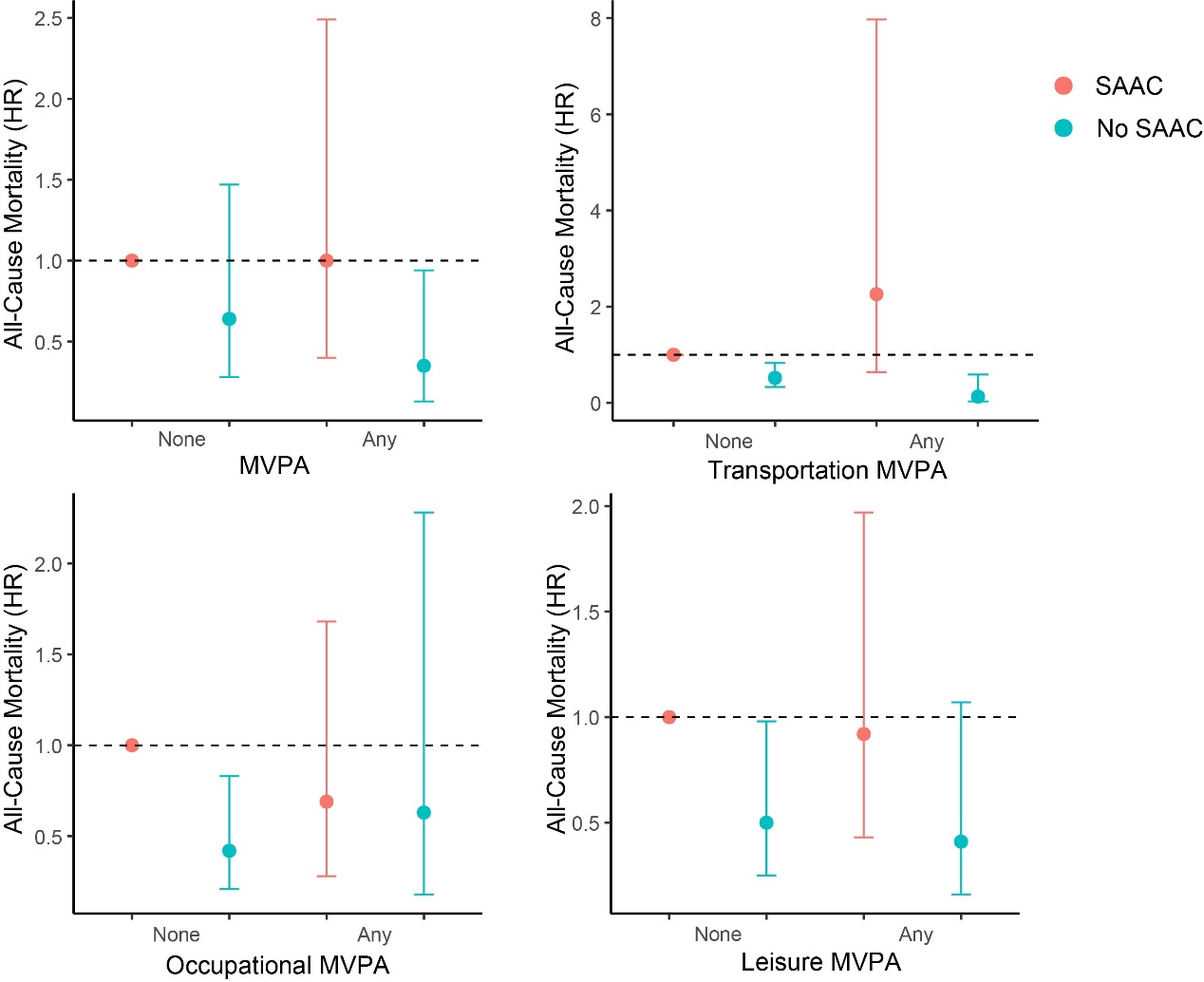


Supplementary Figure S2. The Impact of MVPA on All-Cause Mortality in T2D Grouped by SAAC. MVPA = moderate-to-vigorous physical activity, SAAC = severe abdominal aortic calcification.
